# Youth and family members make meaningful contributions to a randomized‐controlled trial: YouthCan IMPACT

**DOI:** 10.1111/eip.13232

**Published:** 2021-11-01

**Authors:** Joanna Henderson, Lynn Courey, Jacqueline Relihan, Karleigh Darnay, Peter Szatmari, Kristin Cleverley, Amy Cheung, Lisa D. Hawke

**Affiliations:** ^1^ Margaret and Wallace McCain Centre for Child, Youth and Family Mental Health Centre for Addiction and Mental Health Toronto Canada; ^2^ Department of Psychiatry University of Toronto Toronto Canada; ^3^ Sashbear Foundation Toronto Canada; ^4^ Lawrence S. Bloomberg Faculty of Nursing University of Toronto Toronto Canada; ^5^ Sunnybrook Health Sciences Centre Toronto Canada

**Keywords:** engagement, family members, patient‐oriented research, randomized‐controlled trial, youth

## Abstract

**Background:**

There are growing calls to engage service users in research about issues relevant to them. Youth and family members can make meaningful contributions to research projects, improving quality and relevance. However, more information is needed on the contributions that youth and family members can make to various study designs.

**Objective:**

This paper describes the contributions that youth and family members have made to a multi‐site pragmatic randomized‐controlled trial, YouthCan IMPACT, and the way project‐based engagement learnings accelerated change at the institutional level and beyond.

**Results:**

Youth and family members were full members of the project team, including the project's core governance and working groups. They contributed to project leadership, as funding co‐applicants and as equal members of the governance team. They were also engaged in study design. Youth defined the primary outcome measure and contributed to decisions on all secondary measures. The service pathway was co‐designed with youth and family members; for example, they guided the inclusion of peer support and a family member intervention as core service components. Study implementation contributions included ensuring a youth‐ and family‐friendly research process and training research staff on working with youth and family members. Knowledge translation activities have included youth and family members as co‐presenters and manuscript co‐authors. The learnings from this trial have been leveraged to expand youth and family engagement at the institution and beyond.

**Conclusions:**

Youth and family members make substantial contributions to complex research projects, including randomized‐controlled trials, thereby improving project design, study implementation, associated interventions, and knowledge translation.

## 1 BACKGROUND

It is increasingly considered essential to engage individuals with lived experience in all stages of research projects examining issues relevant to them, to improve research quality and relevance (Banner et al., 2019; Bell, 2015; Kirshner et al., 2005; Ontario Centre of Excellence for Child and Youth Mental Health, 2016). Engagement frameworks have been developed, including Canada's Strategy for Patient‐Oriented Research (SPOR; Canadian Institutes of Health Research, 2015), the United Kingdom's National Institute for Health Research (INVOLVE, 2012), and the American Patient‐Centered Outcomes Research Network (Patient‐Centered Outcomes Research Institute, 2018). Integrated knowledge translation is a framework that calls for the active engagement of end‐users and other stakeholders in research to foster uptake (Courtney et al., 2020; Gagliardi et al., 2017).

In research regarding young people, youth engagement is of paramount importance since there can be a gap between the realities facing young people and the experiences of researchers. Youth can be engaged as full partners in research activities (Heffernan et al., 2017; Kirshner et al., 2005). We, as a team, refer to ‘youth engagement’ rather than ‘patient engagement’, as this term is preferred by young people and is more consistent with the framework of engagement; that is, youth do not have to be registered patients of a healthcare institution in order to provide feedback on youth issues and services. In addition to youth engagement, it is also important to engage family members, or caregivers, as they play important roles in treatment decisions (Bannon & McKay, 2005; Logan & King, 2001). Engagement and integrated knowledge translation frameworks recognize the skills and expertise that these important stakeholders can bring to research and decision‐making processes (Gagliardi et al., 2017; Heffernan et al., 2017; Nguyen et al., 2019).

While there is a growing literature describing patient‐led projects, youth‐led projects, participatory‐action research, and qualitative studies (Domecq et al., 2014; Iwasaki et al., 2014; Sarah et al., 2016), there is a paucity of literature on engagement in research designs such as randomized‐controlled trials. It is important to recognize that youth and family members can contribute to a wide variety of study designs, and their involvement should not be limited to certain designs over others. For example, across study designs, they support researchers in identifying research questions and interpretations that address the experiences most relevant to the target population. They can also help ensure that studies are designed in ways that are appealing and experientially appropriate for the target audience, fostering participation, satisfaction, and retention. To support the scale‐up of engagement across areas of research, there have been calls for researchers to share engagement experiences and impacts with research communities (Nguyen et al., 2019).

The YouthCan IMPACT project is a pragmatic randomized‐controlled trial (RCT) and integrated youth service (IYS) development project (Henderson et al., 2017) operating out of the Centre for Addiction and Mental Health (CAMH) in Toronto, Canada. The IYS model is a one‐stop‐shop model of community‐based care for youth wellness, including supports for mental health, substance use, and other areas of wellness. It is an integrated, collaborative care team model bringing together a wide range of interventions and care providers, including social workers, psychiatrists, nurse practitioners, peer support workers, care navigators, and other contributors to the service pathway. IYSs provide a variety of co‐located, evidence‐informed services to meet a wide range of youth needs.

In this complex, multi‐site RCT, youth and family members were engaged throughout, from the grant application to knowledge translation. Prior to funding, youth and family members were consulted to help shape the project. As the YouthCan IMPACT project advanced, a governance model was established; the project was governed by a Core Team, supported by a Community Group to develop the clinical service pathway, a Methods Group to design the study methodology, an Implementation Science Group to develop implementation science assessments and approaches, and a Hospital Group to establish hospital study procedures. A Youth Advisory Group (YAG), Family Member Advisory Committee (FMAC), and a Stakeholder Working Group were also established to support the project.

The McCain Model of Youth Engagement (Heffernan et al., 2017) was developed in concert with the project to guide fulsome engagement in the Margaret and Wallace McCain Centre for Child, Youth and Family Mental Health (McCain Centre), applied directly to the YouthCan IMPACT project. As per the McCain Model, a small number of youth with lived experience of mental health and/or substance use challenges were employed as staff, known as youth engagement facilitators (YEFs). YEFs connected regularly with a broader range of youth with lived experience in a youth advisory group (YAG), as well as a National Youth Action Council (NYAC). One lead family member with lived experience of supporting a youth was a regular team member and co‐investigator, but was not a staff member. Additional family members with family lived experience supported specific processes throughout the study, for example as members of the community working group. The lead family member also connected with a broader group of family representatives in a Family Member Advisory Committee (FMAC). Through these mechanisms, both youth and family members made substantial contributions to the project. Youth and/or family engagement were prioritized for each project activity based on whether the primary end‐user was a youth or family member. By establishing a climate of open, honest, and respectful discussion and building strong relationships, opinions were discussed and consensus was achieved across stakeholder groups through the course of the project. The team's experience of engagement, including the successes and challenges of the engagement process, are described in Sheikhan et al. (2021).

This article describes contributions that youth and family members made to the YouthCan IMPACT project. The purpose is to highlight the ways in which engagement improved the quality of the project, stimulating thought about how youth and family members can contribute to diverse research projects. An overview of youth and family member contributions is provided in Table 1.

**TABLE 1:** Summary of youth and family member contributions to the YouthCan IMPACT project

	Youth	Family members
*Project leadership and vision*		
Overall vision and pre‐award co‐design	✓	✓
Grant co‐applicants	✓	✓
Core team membership for project planning & oversight	✓	✓
Project values development	✓	
*Study design*		
Methods co‐design	✓	
Identification of primary outcome	✓	
Review and co‐select all outcomes and measures	✓	
*Study implementation*		
Review and enhancement of study procedures	✓	✓
Review of scripts & participant materials	✓	
Training of research staff	✓	✓
Participant‐facing study material co‐design	✓	
*Service pathway design*		
Co‐design of service pathway	✓	✓
Creation of youth‐friendly service postcards	✓	
Recommendations on interior design of clinical spaces	✓	
Co‐development of mobile apps list & service provider training	✓	
*Knowledge translation*		
Webinar co‐presenters	✓	✓
Publication co‐authors	✓	✓
Conference co‐presenters	✓	✓
Co‐development of the website	✓	✓

## 2 YOUTH CONTRIBUTIONS

### 2.1 Project leadership and vision

**FIGURE 1:**
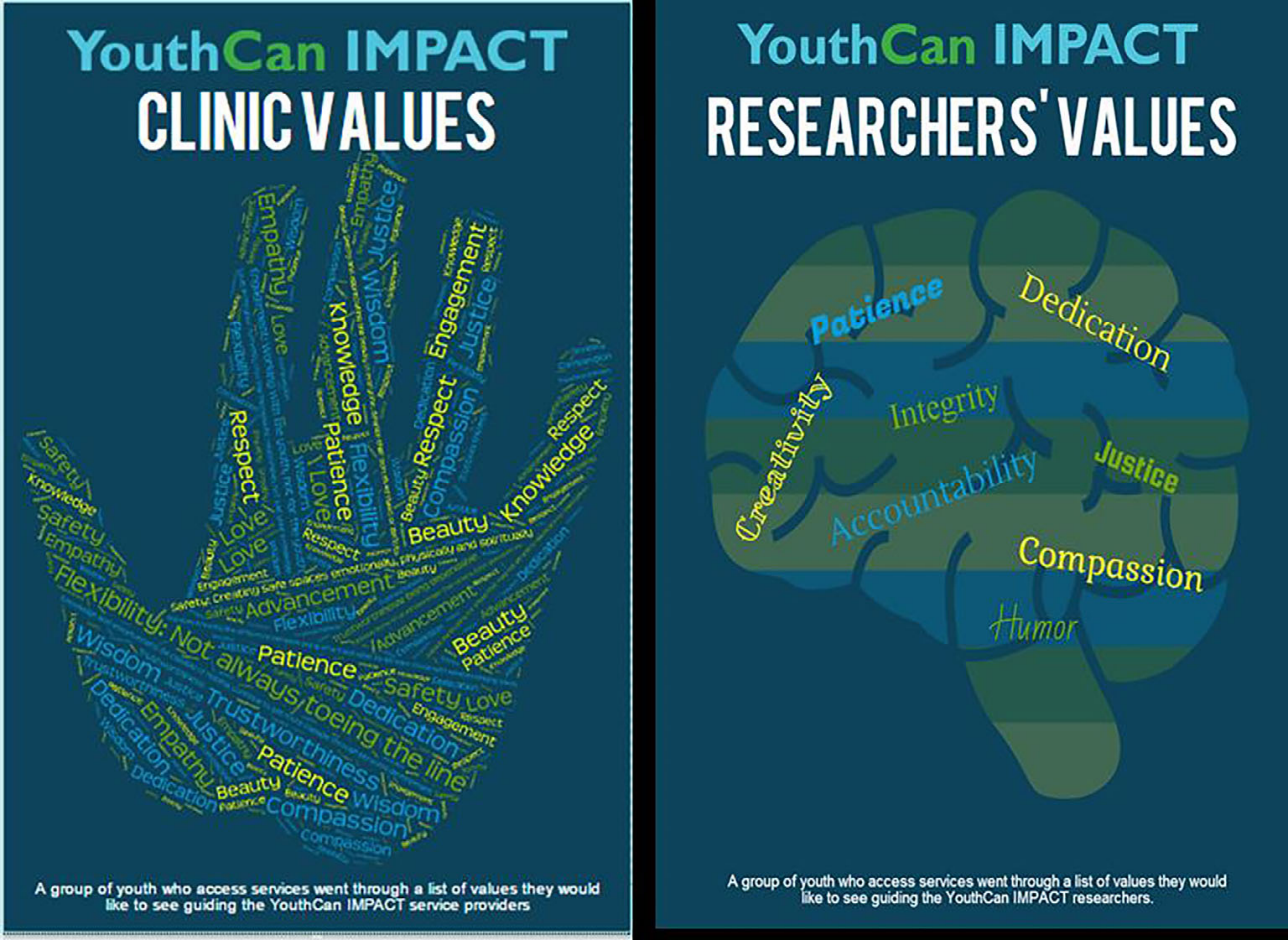
Youth‐generated values underpinning the YouthCan IMPACT project

Prior to obtaining funding and fully implementing the engagement model, ongoing youth consultations helped shape the project vision. Youth were consulted at pre‐funding, when they co‐developed our flexible model of engagement, while also emphasizing the importance youth engagement in the service delivery model via peer support. They also advocated for developing an easy‐to‐access service delivery model that includes crisis supports and holistic supports, building on our vision of what IYSs would offer. Youth were involved in the successful grant application; two YEFs were co‐applicants and helped shape the vision through regular attendance at project design meetings, in conjunction with integration of the recommendations provided during the youth consultations. Once funding was awarded and project work proceeded at a faster pace, YEFs became regular members of the project's Core Team, ensuring that youth voices were paramount during all project discussions. From developing overarching strategies to overseeing timelines, YEFs were present at meetings with the scientists, site representatives and the broad project team. The overarching project vision was enhanced by the YAG members, who developed core values to guide the research and clinical processes and created associated values posters that are prominently displayed in the research and service settings (see Figure 1). These were developed in a YAG meeting in which youth attendees brainstormed together on the values that should drive the project.

### 2.2 Study design

Youth contributed substantially to the research methods development process. In the early development phases, youth defined the primary research outcome—functioning— which they considered more important than diagnostic symptomatology and which makes the study more closely aligned with youth values. YEFs attended regular meetings of the Methods Group, where they provided insights about each potential outcome domain, correlate construct, and measurement tool, ensuring face validity and that measures resonate with participants, which may foster completion. They advocated for the inclusion of secondary outcomes such as empowerment and engagement as participants progressed through the service pathway; this led to the addition of measures for these constructs and will lead to reported outcomes that align with these youth values. The selection of measures occurred through a reciprocal process, where the youth identified outcomes of interest, the researchers identified relevant measures, and potential measures were discussed together among the youth and researchers.

### 2.3 Study implementation

YEFs and YAG members emphasized the importance of ensuring youth‐friendly research processes. They made many suggestions that were incorporated into the study visit procedures, such as offering snacks and activity packages (colouring, word searches, origami), which created a relaxed and less clinical atmosphere. Youth trained the research staff in working with participants in an appropriate, youth‐friendly manner, emphasizing for example the importance of asking which pronouns to use and when (e.g., in contexts with and without family members present), considering potential disability accommodations, youth autonomy in decision‐making, and other important aspects of working with youth. The YEFs played the role of participant in mock participant visits, which supported the staff in developing their communication and interviewing skills and youth‐appropriate language choices. The research staff found these trainings to be highly beneficial, and high satisfaction ratings from youth participants for study visits suggest that this process was successful. The YEFs regularly provided feedback on the wording of study scripts and materials used on a daily basis, attending to reading level and ease of understanding. They also co‐developed participant‐facing study educational materials. Together, these contributions led to a smooth research implementation process, with strong participant retention and minimal challenges to address during the course of the study.

### 2.4 Service pathway design

YEFs were full members of the Community Group, where they contributed directly to the development of the community‐based clinical service pathway. YEFs provided feedback on all of the potential service components and how they could work together. They consistently emphasized the importance of peer support; upon hearing youth perspectives and reviewing the evidence base, both individual and group peer support thereby became core components of the service pathway. YEFs further created postcards describing each main service component in youth‐friendly terms. These postcards are available to any youth accessing the service sites, both within the RCT and via the community. YEFs supported the choice of interior design of the space, for a youth‐friendly setting. In partnership with the research team, YEFs and YAG developed a list of mobile apps to be recommended to service‐seeking youth, a list that is in use at all clinical sites and is freely available online (The YouthCan IMPACT team, 2017). A YEF trained the service providers on the use of these apps. The youth team thereby substantially contributed to designing the youth‐friendly, appropriate service pathway that the study tests.

### 2.5 Knowledge translation

YEFs have been involved in multiple knowledge translation activities, such as co‐authorship on manuscripts (Henderson et al., 2017, 2018), including the current manuscript, co‐presentation at conferences, and co‐facilitation of a project webinar (Cleverley et al., 2016). Through ongoing consultation with the YAG members, the YEFs worked closely with the research team to develop the project website (The YouthCan IMPACT team, 2017) to provide information about the project to a broad audience. Knowledge translation efforts have been enhanced by youth engagement, ensuring that the team continually reports on aspects of the study that are relevant to young people and that diverse audiences are targeted, including youth.

## 3 FAMILY MEMBER CONTRIBUTIONS

### 3.1 Project leadership and vision

During the initial project development phase prior to funding, family members of youth with mental health or substance use challenges were consulted to help shape the project vision. They emphasized the importance of using a one‐stop‐shop team‐based approach; incorporating peer support, care navigation and family member services; and engaging family members throughout the project. For the successful funding application, the lead family member was a co‐investigator and contributed to design discussions. This lead family member was a regular member of the Core Team and ensured that the team kept family member concerns in mind throughout planning.

### 3.2 Study implementation

When planning study implementation, the Family Member Advisory Council (FMAC) emphasized important qualities that research staff must embody to work effectively with youth and family members. For example, they discussed the importance of displaying empathy, true interest in the participant, a validating interpersonal style, confidence in the project, etc. They also pointed to the importance of providing strong staff debriefing sessions. The lead family member and a FMAC representative trained the research staff on working with family members in a validating manner and considering the family member perspective and role, whatever the family dynamic might be. Research staff appreciated this guidance, routinely applied it to participant contacts, and felt that it improved study implementation. Regular clinical research supervision sessions were also established for staff, which were key to smooth study operations. The FMAC also provided valuable feedback on intake processes and helped develop associated procedures. For example, they identified that some families might be disappointed with the randomization results and provided guidance in developing a script for the RAs to manage this situation, which was applied on multiple occasions. Youth and family dyads were recruited via the FMAC for mock study visits prior to study launch, helping to prepare research staff to be confident with their first participants. Their feedback was also considered in developing study scripts. Strong study visit satisfaction rating by family member participants attest to the positive impacts of their contributions.

### 3.3 Service pathway design

Family member representatives were ongoing members of the Community Group, where they co‐created the clinical service pathway. This included the lead family member and other family members over the course of the project. Family members emphasized including a diversity of intervention components. Notably, both the FMAC and the family team members emphasized the importance of providing services for family members, since the wellbeing of family members is intertwined with youth wellbeing. Since the lead family member was a leader of an organization delivering the Family Connections intervention (Courey et al., 2021; Hoffman et al., 2005), which is an evidence‐based program based on the principles of dialectical behavioural therapy and is designed specifically for family members and delivered by family member peers, the lead family member offered to bring this to the service pathway. Family Connections thereby became a core component of the service pathway. In addition to this major contribution, family member representatives provided feedback and guidance in co‐constructing all aspects of the service delivery model.

### 3.4 Knowledge translation

The lead family member was involved in multiple knowledge translation activities, such as manuscript co‐authorship (Henderson et al., 2017; Sheikhan et al., 2021), including the current manuscript, conference presentations, and webinar co‐facilitation (Cleverley et al., 2016). The FMAC contributed to website refinement (The YouthCan IMPACT team, 2017), making sure it was easy to navigate, appealing, and presented information desirable for family members seeking project information.

## 4 PROJECT‐LEVEL ENGAGEMENT AS AN ACCELERATOR OF BROADER SYSTEM CHANGE

By instituting engagement in this single large project and experiencing the benefits of engagement, the team has made gains that has enabled us to integrate engagement at the McCain Centre, at the CAMH organizational level, and beyond.

The lack of a full‐time point‐person dedicated to overseeing engagement was identified as an early barrier, and a Youth Engagement Coordinator was therefore added to the McCain Centre's team. The original Youth Advisory Group developed for YouthCan IMPACT was then expanded to provide Centre‐wide support across a wide range of research projects, clinical activities, and organizational initiatives. The initial team of two YEFs and 11 YAG members supporting YouthCan IMPACT grew to six YEFs, a 15‐member YAG, and a team of 45 youth advisors supporting 25 projects. Multiple YAG members were hired as YEFs over the years, providing opportunities for growth for the engaged youth and for the youth engagement initiative.

The youth engagement team encompasses youth with substantial diversity across mental health and substance use experiences, sociodemographic profiles, and intersectionalities, providing a pool of youth with experience relevant to a wide range of research topics and population subgroups. The team now offers engagement services to other research teams within the McCain Centre and projects conducted in collaboration with McCain Centre scientists. These include research designs such as knowledge synthesis studies (Hawke et al., 2019; Krause et al., 2021), survey studies (Hawke, Barbic, Voineskos, et al., 2020), randomized‐controlled trials (Wiljer et al., 2020), Delphi studies (Cleverley & Rong, 2018), longitudinal studies (Cleverley, Bennett, et al., 2020), discrete choice experiments (Hawke, Hayes, Iyer, et al., 2020), qualitative designs (Cleverley, Lenters, & McCann, 2020), pilot studies (Courtney et al., 2019), clinical pathway development (Courtney et al., 2020), and more, including Centre‐based, province‐wide, and pan‐Canadian studies. The youth team has undertaken a youth‐led research project (Syan et al., 2021) and led components of other projects. They also support multiple knowledge translation initiatives. The referenced materials represent only a small sample of the studies and initiatives that the engagement team is supporting.

At the institutional level, the youth engagement team has helped to develop the first Patient and Family Engagement Roadmap at CAMH based on the McCain Model. The youth engagement team is working with the clinical team to integrate peer support into the clinical services at CAMH and to train both clinicians and research staff on working with youth appropriately in research and service design. The broader team at CAMH is leveraging our engagement experience to develop a CAMH‐wide patient and family engagement strategy; the youth engagement team has supported the development of two patient and family engagement coordinator roles for CAMH, including coordinator and advisor recruitment and researcher training for those roles.

Project‐based engagement learnings have also expanded beyond the organizational level. Notably, the YouthCan IMPACT project inspired the Youth Wellness Hubs Ontario (YWHO) initiative (Youth Wellness Hubs Ontario, 2020), a demonstration project that is scaling the IYS model across Ontario. Leveraging the YouthCan IMPACT engagement experiences, youth and family engagement is a core component and guiding principle of YWHO at the provincial and local levels. The McCain Centre team has also leveraged these learnings to guide national and international researchers on youth engagement. This has included publishing a set of concrete youth engagement recommendations for researchers (Hawke et al., 2018), then partnering with youth, researchers, and community‐based teams across Canada to build a thorough training curriculum (Darnay, Hawke, Chaim, Henderson, & The INNOVATE Research Team, 2019), which describes engagement processes, examples, barriers, facilitators, and mitigating strategies. These materials were used to train researchers across Canada in youth engagement (Hawke, Darnay, Brown, et al., 2020), with the curriculum freely available online. Future research may include an evaluation of the engagement activities.

## 5 CONCLUSIONS

In the growing movement toward engagement in research (Canadian Institutes of Health Research, 2015; INVOLVE, 2012; Patient‐Centered Outcomes Research Institute, 2018), it is important to elucidate the contributions that engaged representatives are making to research projects (Nguyen et al., 2019), illustrating the impacts they can make in a wide variety of research designs. By engaging youth and family members in the YouthCan IMPACT project, youth and family voices were paramount at all stages of the project. Their contributions greatly enhanced the project's quality and relevance to the target population. The learnings from this project were leveraged to expand engagement at the McCain Centre, as well as at the organizational level and beyond. Youth and family members will continue to be engaged as the team concludes the YouthCan IMPACT study, analyzes the data, and reports on the findings.

While a growing body of literature is demonstrating the importance and impact of engagement (Banner et al., 2019; Bombard et al., 2018; INVOLVE, 2012; Laurance et al., 2014; Sheikhan et al., 2021), future research is required to test the impact of engagement models. It is important to continue delineating the role and experience of engagement in diverse research projects as a way to guide researchers in engagement processes (Faithfull et al., 2019; Forsythe et al., 2019; Sheikhan et al., 2021). However, pushing engagement endeavours to a more advanced level requires conducting trials of engagement, explicitly evaluating the impact of engagement on the success of research projects; researchers should consider metrics such as study recruitment and retention success, participant experience, and study outcomes, as well as metrics to be determined in collaboration with engaged individuals. Robust nomenclature and data collection strategies are required to rigorously define the impact of engagement in research on a wide range of metrics. As calls for engagement expand, institutions, research funders, research ethics boards, and peer‐reviewed journals are encouraged to value and require appropriate engagement strategies when making important decisions about research, acknowledging the pivotal role that engaging individuals with lived experience can have in designing and conducting efficient, relevant, and appropriate research projects (Forsythe et al., 2019; Hawke, Darnay, Relihan, et al., 2020).

Youth and family members can make substantial contributions to complex research projects, beyond participatory‐action community‐based designs, including randomized‐controlled trials and other research designs. Robust engagement improves project design, study implementation, associated interventions, and knowledge translation. Researchers are encouraged to consider the impacts that youth and family members can make to their research projects and develop or expand upon their engagement models to optimize the quality of their research work.

## CONFLICT OF INTEREST

The authors declare no conflict of interest.

## FUNDING

Initial funding was provided by the Ontario SPOR SUPPORT Unit, which is supported by the Canadian Institutes of Health Research and the Province of Ontario. Additional funding was provided by the Canadian Institutes of Health Research. The project was also made possible through a financial contribution from the Margaret and Wallace McCain Centre for Child, Youth and Family Mental Health, Centre for Addiction and Mental Health (CAMH). The funders played no role in the design of the project; the collection, analysis, and interpretation of the data, in the writing of the report, or in the decision to submit the paper for publication.

## Data Availability

N/A.
